# Spot urinary sodium in CKD patients: correlation with 24h-excretion and evaluation of commonly used prediction equations

**DOI:** 10.1186/s12882-024-03639-2

**Published:** 2024-06-27

**Authors:** Johanna T. Kurzhagen, Stephanie Titze, Beatrix Büschges-Seraphin, Mario Schiffer, Markus P. Schneider, Kai-Uwe Eckardt, Karl F. Hilgers

**Affiliations:** 1 Department of Nephrology and Hypertension, University of Erlangen-Nuremberg, Medizinische Klinik 4, Universitätsklinikum Erlangen, Erlangen, Germany; 2 Kassenärztliche Bundesvereinigung, Berlin, Germany; 3 KfH Fürth, Nephrology and Hypertension, Fürth, Germany; 4 Department of Nephrology and Medical Intensive Care, Charité – University Medicine Berlin, Berlin, Germany

**Keywords:** 24h urine collection, Spot urine, Electrolytes, Sodium, Chronic kidney disease, Diuretics, Salt

## Abstract

**Background:**

Salt intake in CKD patients can affect cardiovascular risk and kidney disease progression. Twenty-four hour (24h) urine collections are often used to investigate salt metabolism but are cumbersome to perform. We assessed urinary sodium (U-Na) concentration in spot urine samples and investigated the correlation with 24h U-Na excretion and concentration in CKD patients under nephrological care. Further, we studied the role of CKD stage and diuretics and evaluated the performance of commonly used formulas for the prediction of 24h U-Na excretion from spot urine samples.

**Methods:**

One hundred eight patients of the German Chronic Kidney Disease (GCKD) study were included. Each participant collected a 24h urine and two spot urine samples within the same period. The first spot urine sample (AM) was part of the second morning urine. The second urine sample was collected before dinner (PM). Patients were advised to take their medication as usual without changing dietary habits. U-Na concentrations in the two spot urine samples and their average ((AM + PM)/2) were correlated with U-Na concentration and total Na excretion in the 24h urine collections. Correlations were subsequently studied after stratification by CKD stage and diuretic intake. The usefulness of three commonly applied equations to estimate 24h U-Na excretion from spot urine samples (Kawasaki, Tanaka and Intersalt) was determined using Bland–Altman plots, analyses of sensitivity, specificity, as well as positive (PPV) and negative predictive values (NPV).

**Results:**

Participants (42 women, 66 men) were on average (± SD) 62.2 (± 11.9) years old, with a mean serum creatinine of 1.6 (± 0.5) mg/dl. 95% had arterial hypertension, 37% diabetes mellitus and 55% were on diuretics. The best correlation with 24h U-Na total excretion was found for the PM spot U-Na sample. We also found strong correlations when comparing spot and 24h urine U-Na concentration. Correction of spot U-Na for U-creatinine did not improve strength of correlations. Neither CKD stage, nor intake of diuretics had significant impact on these correlations. All examined formulas revealed a significant mean bias. The lowest mean bias and the strongest correlation between estimated and measured U-Na excretion in 24h were obtained using the Tanaka-formula. Also, application of the Tanaka-formula with PM U-Na provided best sensitivity, specificity, PPV and NPV to estimate U-Na excretion > 4g/d corresponding to a salt consumption > 10g/d.

**Conclusion:**

U-Na concentration of spot urine samples correlated with 24h U-Na excretion especially when PM spot U-Na was used. However, correlation coefficients were relatively low. Neither CKD stage nor intake of diuretics appeared to have an influence on these correlations. There was a significant bias for all tested formulas with the Tanaka-formula providing the strongest correlation with measured 24h U-Na excretion. In summary, using spot urine samples together with the Tanaka-formula in epidemiological studies appears feasible to determine associations between approximate salt intake and outcomes in CKD patients. However, the usefulness of spot-urine samples to guide and monitor salt consumption in individual patients remains limited.

**Supplementary Information:**

The online version contains supplementary material available at 10.1186/s12882-024-03639-2.

## Introduction

High salt consumption aggravates arterial hypertension, especially in individuals with impaired kidney function. Patients with chronic kidney disease (CKD) are particularly salt-sensitive [1, 2]. Moreover, independently of blood pressure effects, high salt consumption is associated with increased end-organ damage, including enhanced excretion of albumin [3, 4], progression of CKD, left ventricular hypertrophy and coronary heart disease [5–7].

Given the presumed impact of increased salt intake on health burden and health care costs, efforts are ongoing to reduce salt consumption worldwide. Sodium intake of 1 gram (g) is equivalent to 2.54g salt (sodium chloride) intake. The World Health Organization (WHO) recommends a maximum daily salt intake of 5g [8, 9]. For CKD patients, the restriction of salt intake to 5g/day (d) has been recommended by Kidney Disease: Improving Global Outcomes (KDIGO) [10]. Similarly, for patients with CKD stage G3-5, the National Kidney Foundation Kidney Disease Outcomes Quality Initiative (NKF KDOQI) guideline recommends a sodium intake below 2.3g/d, corresponding to less than 5.8g salt [11]. However, the majority of CKD patients consume higher amounts [12].

The 24h urine collection is considered the gold standard to determine sodium intake [13]. However, it is only used to a limited extent in everyday clinical practice since it is time-consuming and often performed incorrectly, leading to false estimates [14]. Therefore, it would be helpful if the assessment of sodium intake could be simplified by using spot urine samples. Indeed, spot urine samples are already being used in scientific studies to assess salt intake, although this approach has been criticized due to lack of adequate validation [15–21].

Several investigators developed formulas to estimate 24h U-Na excretion from U-Na concentration in spot urine samples. Kawasaki et al. and Tanaka et al. developed formulas based on data from Japanese volunteers, whereas the Intersalt-formula is based on data from Western populations [16, 22, 23]. All studies were designed for individuals with normal kidney function. Application of those formulas in a Korean and US study enrolling CKD patients found limited performance with the Tanaka-formula providing most precise results [24–26].

We aimed to investigate the extent to which U-Na concentrations in spot urine samples correspond to 24h U-Na excretion in Caucasian patients with CKD under nephrological care and to test the usefulness of common equation formulas for 24h U-Na excretion. Our hypothesis was that U-Na from spot urine samples can predict 24h Na excretion in CKD patients. We chose a sub-cohort of the German Chronic Kidney Disease (GCKD) study, to test this hypothesis in a heterogenous cohort under care in an outpatient nephrology practice without limitations regarding medication or nutrition.

## Methods

The current study is a sub-study in 108 participants of the GCKD study, performed in conjunction with the second follow up visit (after two years) in one of the regional study centers at the University Hospital Erlangen. Design and baseline characteristics of the 5217 participants of the GCKD study were published elsewhere [27]. In brief, we enrolled male and female Caucasian CKD patients under nephrological care between 19 and 74 years of age. Inclusion criteria were an estimated glomerular filtration rate (eGFR) of 30-60ml/min/1.73m^2^ or an eGFR > 60ml/min/1.73m^2^ in the presence of albuminuria > 300mg/d or proteinuria > 500mg/d. The GFR was estimated by using the Modification of Diet in Renal Disease- (MDRD-) formula. Exclusion criteria were organ or bone marrow transplantation, active malignancy, heart failure New York Heart Association (NYHA) IV and legal attendance [28].

Informed consent was obtained from all subjects. Subsequently, patients were enrolled in the current sub-study in one outpatient clinic (KfH; Kuratorium für Dialyse und Nierentransplantation Fürth, Germany). The patients were asked explicitly not to change their usual diet or medication during the study.

The GCKD study protocol and the sub-study protocol were approved by the ethics committee at the University of Erlangen-Nuremberg. All data were managed in a Good Clinical Practice (GCP) compliant database by secuTrial® and were pseudonymized.

Patients in the current sub-study were asked to perform a 24h urine collection and to provide two spot urine samples. For the 24h urine collection, the first morning void should be discarded, and study participants documented the time of this void. All urine during the subsequent 24 hours was collected. During these 24h, two timed spot urine specimens were collected in separate containers (125ml). The first spot urine (AM spot urine) was collected from the second morning void, which took place mostly between 8 and 10 AM. Patients were advised to collect the second spot urine sample (PM spot urine) before dinner, mostly between 6 and 8 PM. After completion of all urine samples and the patients’ documentation, the specimens were processed in a standardized fashion. 5ml of the AM and the PM spot urine samples were transferred to collection tubes. The remaining volume of the spot urine samples and the 24h urine collection were merged, and a sample (5ml) from this total volume was transferred into a further collection tube. Additionally, 5ml of the 24h urine collection had to be provided to the treating nephrologist for additional analyses. The remaining volume of the spot urines and the 24h urine collection was measured and rounded up to 50ml steps. The previously removed 20ml were considered negligible. In each of the three urine samples, sodium, potassium and creatinine were analyzed in the Central Clinical Chemistry Laboratory of the University Hospital Erlangen using standard laboratory procedures (U-Na and U-K: Ion Specific Measurement, AU5800, Beckmann/Coulter; U-Crea: photometric, AU5800, Beckmann/Coulter).

The two spot urine samples (AM and PM) met the requirements of the three most common estimation formulas for sodium excretion – Kawasaki, Tanaka and Intersalt [16, 22, 23] with the exception that the PM spot urine sample was not eligible for application of the Kawasaki-formula due to the timing of sampling, as this formula was designed for morning spot urine samples only.

According to the protocol, cases with a 24h urine collection volume below 250ml/d or cases of a collection time below 20h or above 28h were to be excluded. If the collection period differed from 24h within the time window of 20 to 28h, data was corrected to 24h [23].

During the baseline visit of the GCKD study, data on medical history, medication and sociodemographic parameters were collected. Patients were classified as having diabetes with HbA1c ≥ 6.5% and/or being on antidiabetic drugs. Information about the primary underlying kidney disease was provided by the treating nephrologist. For the analysis, underlying diseases were grouped as diabetic nephropathy, vascular nephropathy, glomerular nephropathy, interstitial nephropathy, other diseases/primary cause not to be determined/unknown diseases. At the second follow up visit, which was closest to the time of the current sub-study, three blood pressure measurements were taken every five minutes, and the mean value was calculated (blood pressure monitor: Omron M5 professional HEM-7001-D). The study participants were considered to have arterial hypertension according to the Guidelines of the European Society of Hypertension and the European Society of Cardiology, if the mean of the three blood pressure measurements was ≥ 140mmHg systolic and/or ≥ 90mmHg diastolic and/or at least one antihypertensive drug was taken [29]. At the time of this visit, information on medication was also collected, including different classes of diuretics (potassium-sparing diuretics, thiazide diuretics, aldosterone antagonists, loop diuretics). Moreover, during the follow up visit, blood and urine samples were collected and analyzed centrally for creatinine in serum and urine (enzymatic, Modular (P) Roche), sodium and HbA1c in serum (S-Na: ISE, Modular (ISE) Roche; HbA1c: TINIA, Cobas Integra 400 Plus, Roche), U-albumin/g creatinine (U-albumin: turbidimetry, Modular (P) Roche; U-Crea: enzymatic, Modular (P) Roche). The number of cases differed slightly in individual calculations due to small numbers of missing data.

IBM SPSS Statistics 24 was used for statistical analyses. Data was tested for normal distribution using the Kolmogorow-Smirnow test, Shapiro–Wilk test, and Q-Q curves. If data showed parametric distribution, the mean value and the corresponding standard deviation (SD) are presented. If data revealed non-parametric distribution, median and interquartile range (IQR) are presented.

The correlation between U-Na concentrations of different urine samples was determined using Spearman’s rank correlation coefficient (Spearman's Rho, r). Further, stratifications by a) CKD stage G (“Grade”) according to the KDIGO chronic kidney disease guideline based on patients´ eGFR at the time of the enrollment to the 24h urine sub-study [10] and b) by the intake of diuretics (yes/no) was performed. The correlation coefficients in independent samples were compared by the Fisher Z value. If the *p*-value was < 0.05, the results were considered statistically significant. For the evaluation of existing estimation formulas, the correlations between measured and calculated sodium excretion values were analyzed and biases were studied using Bland–Altman plots [30, 31]. In addition, the corresponding 95% confidence interval (CI =  ± 1.96 standard deviation (± 1.96 SD)) and ± 30%-precision was calculated. In order to assess a salt intake per day above or below 5g/d, as recommended by WHO, or salt intake above or below 10g/d, sensitivity, specificity, positive predictive value (PPV) and negative predictive value (NPV) of the estimation formulas to predict a U-Na excretion > 2g/d or > 4g/d was determined by using different spot urine samples [8] (Table 1).

**Table 1:** Common equations (Kawasaki- [22], Tanaka- [16] and Intersalt-formula [23]) to predict 24 h U-Na excretion using U-Na concentrations of spot urine samples

Kawasaki-equation for sodium	
**Men**	${\varvec{M}}e24hUNa \left(\frac{mg}{d}\right)=23\times \Big[\!\!\Big[\text{16,3}\times {\left\{\left(\frac{SpotUNa \left(\frac{mmol}{l}\right)}{SpotUCrea\left(\frac{mg}{dl}\right)\times 10}\right)\times \left(15.12\times weight \left(kg\right)+7.39\times height \left(cm\right)-12.63\times age \left(years\right)-79.9\right)\right\}}^{0.5}\Big]\!\!\Big]$
**Women**	${\varvec{W}}e24hUNa \left(\frac{mg}{d}\right)=23\times \Big[\!\!\Big[\text{16.3}\times {\left\{\left(\frac{Spot\text{U}Na \left(\frac{mmol}{l}\right)}{SpotUCrea\left(\frac{mg}{dl}\right)\times 10}\right)\times \left(8.58\times weight\ (kg) +5.09\times height \left(cm\right)-4.72\times age \left(years\right)-74.5\right)\right\}}^{0.5}\Big]\!\!\Big]$
**Tanaka-equation for sodium**	
**Men+Women**	$e24hUNa \left(\frac{mg}{d}\right)=23\times \left[21.98\times {\left\{\left(\frac{SpotUNa\left(\frac{mmol}{l}\right)}{SpotUCrea\left(\frac{mg}{dl}\right)\times 10}\right)\times \left(\left(-2.04\times age \left(years\right)\right)+\left(14.89\times weight \left(kg\right)\right)+\left(16.14\times height \left(cm\right)\right)-2244.45\right)\right\}}^{0.392}\right]$
**Intersalt-equation for sodium**	
**Men**	${\varvec{M}}e24hUNa \left(\frac{mg}{d}\right)=23\times \left\{25.46+\left(0.46\times SpotUNa\left(\frac{mmol}{l}\right)\right)-\left(2.75\times Spot\text{U}Crea\left(\frac{mmol}{l}\right)\right)-\left(0.13\times SpotUK\left(\frac{mmol}{l}\right)\right)+\left(4.10\times BMI\left(\frac{kg}{{m}^{2}}\right)\right)+\left(0.26\times age \left(years\right)\right)\right\}$
**Women**	${\varvec{W}}e24hUNa \left(\frac{mg}{d}\right)=23\times \left\{5.07+\left(0.34\times SpotUNa\left(\frac{mmol}{l}\right)\right)-\left(2.16\times SpotUCrea\left(\frac{mmol}{l}\right)\right)-\left(0.09\times SpotUK\left(\frac{mmol}{l}\right)\right)+\left(2.39\times BMI\left(\frac{kg}{{m}^{2}}\right)\right)+\left(2.35\times age \left(years\right)\right)-\left(0.03\times {age}^{2}\left(years\right)\right)\right\}$
**Sensitivity and specificity**	
$\text{sensitivity}:\frac{correct\ e24hUNa>2 or 4g/d}{m24hUNa>2 or 4g/d}\times 100$ $\text{specificity}: \frac{correct\ e24hUNa<2 or 4g/d}{m24hUNa<2 or 4g/d}\times 100$ $\text{PPV}: \frac{correct\ e24hUNa>2 or 4g/d}{e24hUNa>2 or 4g/d}\times 100$ $\text{NPV}:\frac{correct\ e24hUNa<2 or 4g/d}{e24hUNa<2 or 4g/d}\times 100$	

Me24hUNa = equation for men to predict 24 h U-Na excretion, We24hUNa = equation for women to predict 24 h U-Na excretion, e24hUNa = equation for men and women to predict 24 h U-Na excretion, SpotUNa = U-Na concentrations in spot urine sample, SpotUCrea = U-creatinine concentrations in spot urine sample, SpotUK = U-potassium concentrations in spot urine sample, BMI = Body Mass Index, correct e24hUNa > 2 or 4 g/d = equation correctly predicted 24 h U-Na excretion > 2 or 4 g/day, correct e24hUNa < 2 or 4 g/d = equation correctly predicted 24 h U-Na excretion < 2 or 4 g/day, m24hUNa > 2 or 4 g/d = measured 24 h U-Na excretion > 2 or 4 g/day, m24hUNa < 2 or 4 g/d = measured 24 h U-Na excretion < 2 or 4 g/day, e24hUNa > 2 or 4 g/d = equation predicted 24 h U-Na excretion > 2 or 4 g/day, e24hUNa < 2 or 4 g/d = equation predicted 24 h U-Na excretion < 2 or 4 g/day PPV = positive predictive value, NPV = negative predictive value

## Results

Of the 108 patients enrolled in the study, 66 were men (61%) and 42 women (39%). The average age was 62.2 years (SD 11.9 years) and the mean BMI was 29.7 kg/m^2^ (SD 6.2 kg/m^2^). More than one third of the participants (n = 40; 37%) had diabetes mellitus and the majority was hypertensive (n = 103; 95%).

The serum creatinine value averaged 1.56mg/dl (SD 0.5mg/dl), corresponding to an eGFR of 45.6ml/min/1.73m^2^ (SD 16.9ml/min/1.73m^2^) using MDRD formula. The median urinary albumin excretion as measured in spot urine samples was 20mg/g Creatinine (IQR 4–168 mg/g Creatinine).CKD stages and corresponding risk categories according to KDIGO are presented in supplement table S1 [32]. Individuals with CKD stages outside the range of GCKD inclusion criteria, i.e. eGFR above or below 30–60 mL/min/1.73m^2^ reflect changes in eGFR during the approximately two years since patient screening. The mean serum sodium was 145mmol/l (SD 4.8mmol/l).

The underlying kidney diseases of the study participants reported by the treating nephrologist are summarized in supplement table S2.

Fifty-nine participants (54.6%) were treated with at least one diuretic, most frequently with loop diuretics (33.3%), followed by thiazide diuretics (29.6%), potassium-sparing diuretics (7.4%) and aldosterone antagonists (5.6%). 19% took more than one diuretic from different classes.

The mean urine collection time was 24.2h (± 1h) with a range from 20.8h to 26.7h. The mean urine volume was 2511ml, with a range from 500 to 6500ml. Thus, according to the pre-specified thresholds no study participants had to be excluded, because neither urine volume nor sampling time were outside the pre-defined tolerance range.

### Correlations between U-Na concentrations of AM and PM spot urine sample with 24h U-Na total excretion

**Fig. 1:**
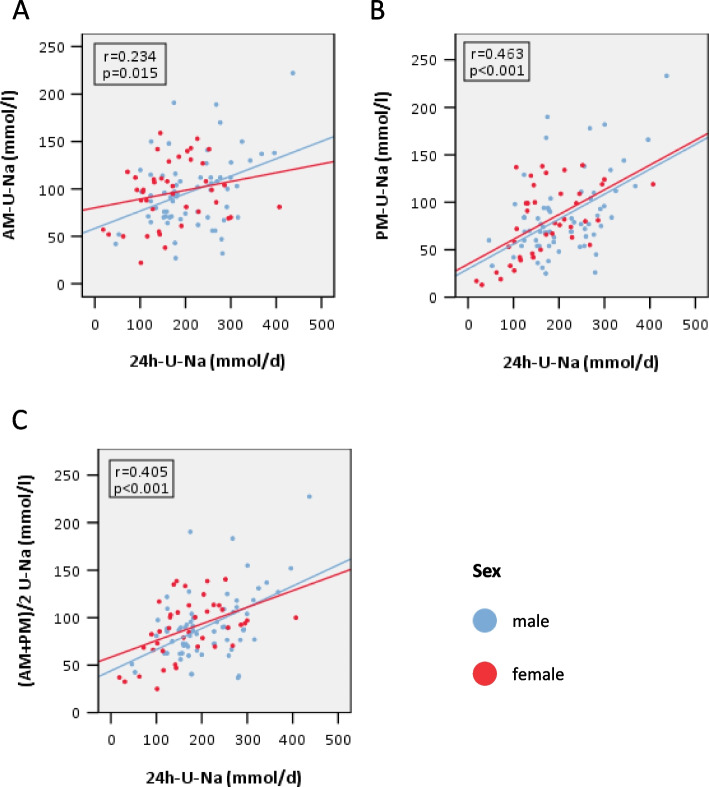
Correlation of U-Na concentrations of different spot urine samples with 24h U-Na excretion (r = Spearman´s Rank correlation coefficient) **A** Correlation AM U-Na concentration (mmol/l) with 24h U-Na excretion (mmol/d), AM = morning spot urine sample (second morning urine), n = 108, r = 0.234, *p* = 0.015. **B** Correlation PM U-Na concentration (mmol/l) with 24h U-Na excretion (mmol/d), PM = evening spot urine sample, n = 107, r = 0.463, *p* < 0.001. **C** Correlation (AM + PM)/2 U-Na concentration (mmol/l) with 24h U-Na excretion (mmol/d), (AM + PM)/2 = Average of morning and evening spot urine, n = 107, r = 0.405, *p* < 0.001; *U-Na* = *urine sodium, blue: male study participants, red: female study participants*

Figure 1 shows the correlations of U-Na concentrations in AM and PM spot urine samples and their average (AM + PM)/2 with measured U-Na excretion in 24h urine collection (mmol/l in spot urine vs. mmol/d in 24h urine samples). The numerically strongest correlation was found for PM vs. 24h urine excretion followed by (AM + PM)/2 vs. 24h urine excretion and AM vs. 24h urine excretion. Accordingly, there was less scatter for U-Na concentrations in PM and average (AM + PM)/2 vs. 24h urine U-Na compared to U-Na in AM spot urine samples. The correlation of AM vs. 24h urine and (AM + PM)/2 vs. 24h urine (Z-value: -1.382, *p* = 0.083) was comparable. AM vs. 24h urine and PM vs. 24h urine were significantly different (Z-value: -1.899, *p* = 0.029). The comparison of PM vs. 24h urine and (AM + PM)/2 vs. 24h urine excretion did not show statistically significant differences (Z-value: -0.516, *p* = 0.303) (Fig. 1, Table 2).

**Table 2:** Correlation U-Na concentration in different spot urine samples (mmol/l) vs. 24 h U-Na excretion (mmol/d) and stratification based on CKD stage and intake of diuretics

	AM (mmol/l) vs. 24h (mmol/d)		PM (mmol/l) vs. 24h (mmol/d)		(AM + PM)/2 (mmol/l) vs. 24h (mmol/d)	
All	r = 0.234 (*p* = 0.015) n = 108		**r = 0.463** (*p* < 0.001) n = 107		r = 0.405 (*p* < 0.001) n = 107	
CKD stage	G1-3a	G3b-5	G1-3a	G3b-5	G1-3a	G3b-5
	r = 0.201 (*p* = 0.165) n = 49	r = 0.328 (*p* = 0.011) n = 59	r = 0.444 (*p* = 0.001) n = 49	r = 0.509 (*p* < 0.001) n = 58	r = 0.358 (*p* = 0.012) n = 49	r = 0.444 (*p* < 0.001) n = 58
	Z = -0.688 *p* = 0.246		Z = -0.421 *p* = 0.337		Z = -0.514 *p* = 0.304	
Diuretics	ND	D	ND	D	ND	D
	r = -0.033 (*p* = 0.821) n = 49	r = 0.447 (*p* < 0.001) n = 59	r = 0.370 (*p* = 0.009) n = 49	r = 0.5 (*p* < 0.001) n = 58	r = 0.218 (*p* = 0.132) n = 49	r = 0.531 (*p* < 0.001) n = 58
	Z = -2.583 *p* = 0.005		Z = -0.805 *p* = 0.210		Z = -1.852 *p* = 0.032	

AM urine = morning spot urine, PM urine = evening spot urine, ((AM + PM)/2) urine = average morning + evening spot urine, 24h urine = 24h urine collection test, U = urine, ND = no intake of diuretics; D = intake of diuretics; bold print is the highest correlation coefficient (Spearman's Rho, r)

**Fig. 2:**
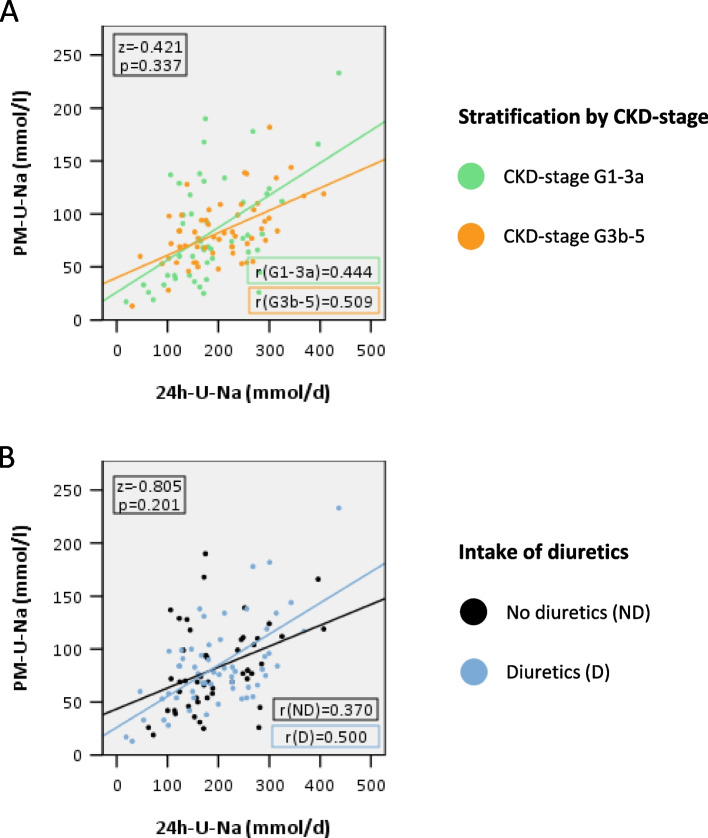
Comparison of correlations by CKD stage and intake of diuretics (mmol/l vs. mmol/d). **A** Correlation PM U-Na concentration (mmol/l) with 24h U-Na excretion (mmol/d) by CKD stage (G1-3a vs. G3b-5) n(G1-3a) = 49, n(G3b-5) = 58, r(G1-3a) = 0.444, r(G3b-5) = 0.509, z = 0.421, *p* = 0.337; *p* > *0.05 and the differences are therefore not significant; green: mild CKD* = *CKD stage G1-3a, orange: moderate CKD* = *CKD stage G3b-5.* **B** Correlation PM U-Na concentration (mmol/l) with 24h U-Na excretion (mmol/d) by intake of diuretics. n(ND) = 49, n(D) = 58, r(ND) = 0.370, r(D) = 0.500, z = -0.805; *p* > *0.05 and the differences are therefore not significant; black: no intake of diuretics (ND), blue: intake of diuretics (D). CKD* = *chronic kidney disease; U-Na* = *Urine sodium, r = Spearman´s ranking correlation coefficient, (AM + PM)/2 = average morning and evening spot urine; ND = no intake of diuretics; D = intake of diuretics*

Figure 2A shows the correlation between U-Na concentration of spot urine samples PM and 24h U-Na excretion by CKD stage (G1-G3a vs. G3b-G5). The correlations were similarly strong for both groups with no statically significant differences (CKD stage 1-3a: r = 0.444, *p* < 0.001 vs. CKD stage 3b-5: r = 0.509; *p* < 0.001; Z-value = 0.421, *p* = 0.337) (Table 2).

Also, no significant difference was found between correlations in study participants who took diuretics and those who did not (no diuretics: r = 0.370, *p* = 0.009 vs. diuretics: r = 0.500, *p* < 0.001; Z-value = -0.805, *p* = 0.210) (Fig. 2B, Table 2).

### Correlations between U-Na concentration of AM and PM spot urine sample with 24h U-Na concentration

We found strong correlations also when comparing U-Na concentrations in spot urine samples with U-Na concentrations in 24h urine collections (mmol/l in spot urine vs. mmol/l in 24h urine samples). Here, AM showed the weakest correlation. (AM + PM)/2 was superior to PM in this evaluation, but with minor difference (supplement figure S1). Stratification by CKD stage and diuretic use revealed no significant differences between groups (supplement figure S2 and supplement table S3).

### Adjustment to U-creatinine

We next analyzed whether adjustment to U-creatinine concentrations would further improve the associations. However, the correlations between U-Na/g creatinine (mmol/g creatinine) in different spots urine samples and 24h U-Na excretion (mmol/d) were less strong than correlations without adjustment for creatinine excretion. Here, PM also showed a slightly stronger correlation than (AM + PM)/2 with 24h U-Na excretion. Again, neither the intake of diuretics nor CKD stage had significant impact on these correlations (supplement table S4).

### Assessment of estimation formulas

When applying different formulas (Table 1) to estimate 24h U-Na excretion from U-Na concentrations in spot samples, significant positive correlations as well as a significant mean biases were found (supplement table S5). The strongest correlation was found when using the Tanaka-formula with the PM spot urine concentration (r = 0.533, *p* < 0.05). The calculations using the Intersalt-formula also revealed significant, albeit weaker correlations (PM: r = 0.469, *p* < 0.05; AM: r = 0.403, *p* < 0.05). The correlations between the measured U-Na excretion in 24-h urine and the results of the Kawasaki-formula were weaker (Kawasaki AM: r = 0.393, *p* < 0.05). The weakest correlation was found with application of the Tanaka-formula to the AM-spot urine concentrations (r = 0.375, *p* < 0.05). However, there were no statistically significant differences between any of those correlations (supplement figure S3, supplement table S5).

**Fig. 3:**
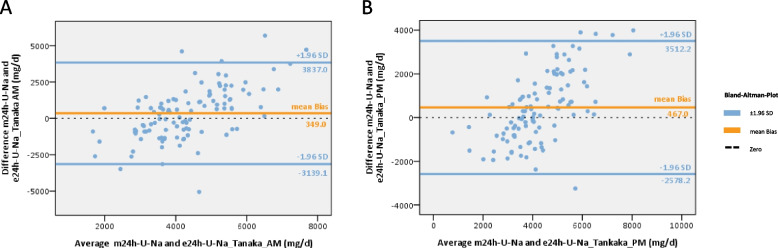
Assessment of Tanaka-equation to predict 24h U-Na excretion from AM and PM U-Na concentrations. **A** Bland–Altman plot using the Tanaka-formula and AM spot urine, n = 108, mean bias (± 1.96 SD) = 349 (-3139.1–3837.0). **B** Bland–Altman plot using the Tanaka-formula and PM spot urine, n = 107, mean bias (± 1.96 SD) = 467.0 (-2578.2–3512.2). *m24h-U-Na* = *measured 24h U-Na excretion, e24h-U-Na* = *estimated 24h U-Na excretion, orange line: mean bias, blue line:* ± *95% confidence interval* =  ± *1.96 standard deviation (*± *1.96 SD) of mean bias*

**Fig. 4:**
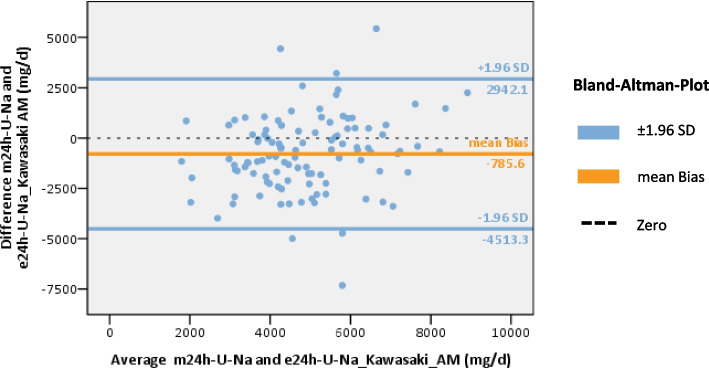
Assessment of Kawasaki-equation to predict 24h U-Na excretion from AM U-Na concentrations. Bland–Altman plot using Kawasaki-formula and AM spot urine, n = 108, mean bias (± 1.96 SD) = -785.6 (-4513.3–2942.1). *m24h-U-Na* = *measured 24h U-Na excretion, e24h-U-Na* = *estimated 24h U-Na excretion, orange line: mean bias, blue line:* ± *95% confidence interval* =  ± *1.96 standard deviation (*± *1.96 SD) of mean bias*

**Fig. 5:**
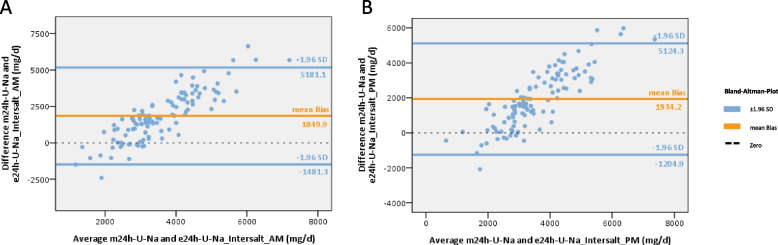
Assessment of Intersalt-equation to predict 24h U-Na excretion from AM and PM spot urine samples. **A** Bland–Altman plot using the Intersalt-formula and AM spot urine, n = 108, mean bias (± 1.96 SD) = 1849.9 (-1481.3–5181.1). **B** Bland–Altman plot using the Intersalt-formula and PM spot urine, n = 107, mean bias (± 1.96 SD) = 1934.2 (-1204.9–5124.3). *m24h-U-Na* = *measured 24h U-Na excretion, e24h-U-Na* = *estimated 24h U-Na excretion, orange line: mean bias, blue line:* ± *95% confidence interval* =  ± *1.96 standard deviation (*± *1.96 SD) of mean bias*

The mean bias was lowest when using the Tanaka-formula (mean bias AM: + 349.0mg U-Na/d, PM: + 467.0mg U-Na/d) (Fig. 3). While the mean bias was lower when using the AM-spot urine samples, the PM-spot urine samples showed a stronger correlation and a narrower 95% CI (Fig. 3, supplement table S5). The results of the Kawasaki-formula showed a slightly higher mean bias (AM: -785.6mg U-Na/d) (Fig. 4). The estimates using the Intersalt-formula differed significantly from the measured values (mean bias AM: + 1849.9mg U-Na/d, PM: + 1934.2mg U Na/d) (Fig. 5). U-Na excretion tended to be underestimated by the Tanaka- and Intersalt-formulas, but was overestimated by the Kawasaki-formula. All formulas tended to underestimate at higher U-Na and overestimate at lower 24h U-Na excretions. All formulas tested showed wide 95%-CI (Figs. 3–5). When assessing sensitivity, specificity, PPV and NPV of different equations in estimating U-Na excretion > 2g/day (corresponding to a salt intake of > 5g/day) the Intersalt-equation using PM spot urine specimen performed best in sensitivity: 96.% and specificity: 100%. However, NPV was low with 60% (Table 3). Since the average salt intake of CKD patients in general and in the studied cohort tends to be higher, we also assessed performance of different formulas for a threshold of > 4g sodium excretion (corresponding to a salt intake of > 10g/day). Here, the Tanaka-equation provided best results (see Table 3).

**Table 3:** Sensitivity, specificity, PPV and NPV of common equations in predicting 24 h U-Na excretion > 2 g/day (salt intake > 5 g/day) or 24 h U-Na excretion > 4 g/day (salt intake > 10 g/day). PPV = positive predictive value, NPV = negative predictive value

			**Sensitivity (%)**	**Specificity (%)**	**PPV (%)**	**NPV (%)**
**Salt intake > 5 g/day**	**Tanaka-equation**	**AM**	100.0	40.0	97.1	100.0
		**PM**	100.0	60.0	98.1	100.0
	**Kawasaki-equation**	**AM**	100.0	16.7	95.3	100.0
	**Intersalt-equation**	**AM**	93.1	100.0	100.0	46.2
		**PM**	96.0	100.0	100.0	60.0
**Salt intake > 10 g/day**	**Tanaka-equation**	**AM**	100.0	90.0	92.1	100.0
		**PM**	86.0	100.0	100.0	86.2
	**Kawasaki-equation**	**AM**	100.0	48.0	69.0	100.0
	**Intersalt-equation**	**AM**	1.7	100.0	100.0	46.7
		**PM**	1.8	100.0	100.0	47.2

To gain a deeper insight in the performance of the Tanaka-formula and assess its potential clinical applicability in individual patients, ± 30%-precision was calculated – a broadly used statistical approach [33]. ± 30%-precision was 56% for the Tanaka-formula using AM spoturine and 54% using PM spot urine.

## Discussion

Regular monitoring of salt intake is particularly important for CKD patients due to higher rates of salt sensitive arterial hypertension and related risks for cardiovascular complications and progression of kidney disease. While the 24h urine collection is considered the gold standard for determining salt intake, this method lacks practicability and is therefore avoided by patients and health care providers [13].

In this study, 108 Caucasian CKD patients under regular nephrological care were investigated to determine whether spot urine samples can replace the 24h urine collection for assessment of total sodium excretion as a putative surrogate for salt intake [27, 34]. While we found fairly good correlations between spot U-Na concentrations and 24h U-Na excretion for all spot urine samples examined, the PM spot U-Na concentration provided best results in this respect with strongest correlations with 24h U-Na excretion.

Similar results were observed in a study cohort from the US (adjusted values: PM: r = 0.86; AM: r = 0.31), which had comparable proportions of hypertensive patients and patients taking diuretics. However, study participation in this study was not limited to CKD patients [35]. Han et. al also found similar results when examining Chinese hypertensive subjects without CKD (PM: r = 0.406; AM: r = 0.057) [36].

When comparing strength of correlations between spot U-Na concentration and 24h U-Na excretion in CKD cohorts, the advantage of PM over AM spot urine samples has been previously shown in a Korean study [17]. Additionally, strongest correlations were found when using the average of two timed spot urine specimens, which we also observed in our study, where mean values of the two spot urine samples provided better results than AM or PM spot urine separately. The authors of the Korean study speculate that the mean of the two spot urine samples averages out circadian variations. The superiority of PM vs. AM spot urine might be due to U-Na concentration in CKD patients peaking in the evening according to these authors [17].

Doenyas-Barak et al. made similar observations in 50 young healthy subjects from Israel who were taking neither diuretics nor antihypertensives. Comparing average values of two, three and four spot urine samples, the results were best for the average of the four spot urine samples. However, the average of two samples was also superior to individual samples [19].

Yet, comparison of different studies is only possible to a limited extent since many study details were different across the studies, such as timing of collection, nutrition, medication and physical activity of patients, as well as ethnic origin of participants [20].

In our study correction for U-creatinine concentration did not improve the correlation with 24h U-sodium excretion. Kang et al. and Mann et al. previously made similar observations [17, 35]. It was assumed that U-creatinine excretion changes due to interindividual variation such as muscle mass, sex, timing of food intake, exercise and incomplete collection time. Mann et al. were able to improve correlations when a correction for 24h U-creatinine excretion was performed [35]. However, this approach brings no advantage since it requires a 24h urine collection itself. Thus, correction for U-creatinine is not recommended when measuring U-Na concentrations in spot urine samples to estimate daily U-Na excretion.

Interestingly, neither CKD stage nor use of diuretics had a relevant impact on any of the tested correlations. Mann et al. also tested the influence of diuretic intake and did not find a significant impact on correlations [35]. We assume that most of their and our study participants had impaired kidney function on a relatively stable level. It has been described elsewhere that in fact disturbance of electrolyte excretion in CKD patients can reach a stable level [37].

To compare the performance of commonly used formulas (Tanaka, Kawasaki and Intersalt), we used Bland–Altman´s approach. Therefore, correlations between measured and estimated 24h U-Na excretion were plotted and the difference between two test results was plotted against the average of the two results. Moderately strong correlations between measured and calculated U-Na excretion were found. The best results between measured and calculated 24h U-Na excretion with the lowest bias were obtained using the Tanaka-formula.

Previous studies comparing different formulas in CKD patients and patients taking diuretics also found the Tanaka-formula to be superior to others [24–26]. However, this was not the case when the equations were applied in healthy cohorts. Here, the Intersalt-formula showed best results [38–40]. Ogura et al. tested the practicability of the Tanaka-formula in CKD patients and found the best correlation with least interquartile range for CKD stages G4 and G5 and for patients with a higher salt consumption. The authors attributed this to the altered circadian rhythm of sodium excretion during progression of CKD [24]. In fact, Tanaka et al. also showed that their formula performed better in individuals with a relatively high salt intake, as it was the case with the original cohort. The study cohort of Tanaka et al. had a mean salt intake of approximately 11.2g/d [16]. The average salt intake of patients participating in our GCKD-24h urine sub-study was similarly high with 10.7g/d, providing a possible explanation for fairly good results of the Tanaka-formula.

Our Bland-Altmann-plots further show that application of the Kawasaki-equation tends to underestimate 24h Na-excretion while the Tanaka- and the Intersalt-formula tend to overestimate 24h Na-excretion. Similar results were reported by Polonia et al. when assessing a Portuguese cohort [41].

Since Bland–Altman plots, which we and others used, assess agreement between two methods, but not the accuracy we also assessed sensitivity and specificity.

All tested formulas showed sensitivity > 93% when predicting whether salt intake was above or below 5g per day. However, the specificity of different formulas was much more variable. While the specificity of the Intersalt-formula was 100%, the Tanaka - and the Kawasaki-equation displayed specificity values below 60%. Moreover, the high specificity of 100% of the Intersalt-formula application comes at the cost of the strong tendency to underestimate U-Na excretion. Accordingly, the Intersalt-formula provided very low NPVs. In a meta-analysis, Huang et al. also demonstrated a very good overall sensitivity (97%) and specificity (100%) of different estimation formulas to predict whether the salt consumption of a population group was above or below the threshold of 5g. However, positive and negative predictive values were not reported [42].

Taking into account that the average salt intake of our CKD cohort was above 10g per day, we also analyzed performance of different estimation formulas to predict a salt intake above 10g per day. Here, the Tanaka formula provided satisfying results. On the other hand, the Intersalt-equation resulted in very low sensitivity (1.8%) given the tendency of underestimation in individuals with higher sodium excretion (Fig. 5).

For the Tanaka-formula, ± 30%-precision was 56% using AM and 54% using PM-spot urine indicating that an application of this formula on an individual level is limited.

Our study has some limitations. First, we took only two different spot urine samples per day and did not investigate differently timed spot urine samples. Thus, we did not test whether the average U-Na of several spot urine samples improves prediction, as others have proposed [43]. Second, we confirmed the use of formulas to predict salt excretion in CKD patients to those best established and did not test others [44–46].

Third, although we have assessed the validity of formulas to estimate 24h U-Na excretion, we have neither estimated sodium intake from the ingested food nor used a defined sodium intake as we were primarily interested in real world applicability. However, previous studies have confirmed that 24h urine U-Na correlated well with the salt intake estimated using nutrition questionnaires [17].

Nevertheless, the validity of 24h U-Na excretion as an indicator of salt intake must be considered. Large *inter*individual fluctuations in sodium excretion, partly independent of salt intake have been reported [15, 47, 48]. U-Na excretion has also been shown to be subject to large *intra*individual fluctuations [15, 49]. Known influencing factors, in addition to nutrition, are variable losses via saliva, the intestine and sweat [50]. While we did not measure *intra*individual variability, the participants of our study showed a wide, more than 20-fold range of U-Na excretion in 24h urine (minimum 19mmol/d, maximum 437mmol/d), which is consistent with previously reported variability. There are also indications that the dynamics of U-Na excretion in healthy individuals differ from those of CKD patients [51]. In addition to fluctuations caused by external factors, an infradiane rhythm has been described that is independent of salt consumption and is based on the release of aldosterone and cortisone [52].

Finally, previous investigations have shown that 24h U-Na excretion is not able to provide a full insight into the complexity of salt metabolism. Day-to-day changes of salt intake may impact the results [52]. There is evidence that not only salt consumption, but also the distribution of sodium in the body is important in the development of essential hypertension [53], which can be assessed by the novel sodium magnet resonance imaging (MRI). In patients with arterial hypertension, sodium storage in the skin is increased compared to normotensive subjects [54]. However, more extensive examination methods including urine collections over several days do not provide practicable alternatives to the 24-h urine collection, especially for large study collectives or in everyday clinical practice. Spot urine specimens on the other hand are inexpensive bio samples that are easy to obtain.

## Conclusion

Measurement of U-Na concentrations in spot urine samples of patients with mild to severe CKD correlate with measurements of 24h U-Na excretion with no improvement through correction for U-creatinine concentrations. However, correlation coefficients were relatively low. Therefore, U-Na in spot urine does not sufficiently predict 24h U-Na excretion to assess salt intake in individuals in clinical praxis. However, spot urine samples together with the Tanaka-formula in epidemiological studies appear feasible to determine associations between approximate salt intake and outcomes.

### Supplementary Information

Supplementary Material 1.

Supplementary Material 2.

Supplementary Material 3.

Supplementary Material 4.

## Data Availability

The data that support the findings of this study are available from Department of Nephrology of University Hospital Erlangen but restrictions apply to the availability of these data, which were used under license for the current study, and so are not publicly available. Data are however available from the authors upon reasonable request and with permission of the German Chronic Kidney Disease committee. To request the data from this study please contact Dr. Johanna Kurzhagen, Medizinische Klinik 4, University hospital Erlangen, Ulmenweg 18, 91054 Erlangen or via johanna.kurzhagen@uk-erlangen.de.

## Abbreviations

A: Albuminuria
AM spot urine: Ante Meridiem, morning spot urine, second morning urine (08:00–10:00 AM)
$\frac{\text{AM}+\text{PM}}{2}\text{ spot urine}$: Average of AM and PM spot urine
BMI: Body Mass Index
CI: Confidence Intervall
CKD: Chronic Kidney Disease
correct e24hUNa > 2g/d: Equation correctly predicted 24h-urine-sodium > 2g/day
correct e24hUNa < 2g/d: Equation correctly predicted 24h-urine-sodium < 2g/day
D: Diuretic intake
eGFR: Estimated glomerular filtration rate
e24hUK: Equation for men and women to predict 24h-urine potassium
e24hUNa: Equation for men and women to predict 24h-urine sodium
G: eGFR
GCKD Study: German Chronic Kidney Disease Study
GCP: Good Clinical Practice
HbA1c: Glycated hemoglobin
Intersalt: International Cooperative Study on Salt and Blood Pressure
ISE: Ion selective electrode
IQR: Interquartile Range
KDIGO: Kidney Disease: Improving Global Outcomes
KfH: Kuratorium für Dialyse und Nierentransplantation, dialysis practice
MRI: Magnet resonance imaging
Me24hUK: Equation for men to predict 24h-urine-potassium
Me24hUNa: Equation for men to predict 24h-urine-sodium
MDRD-formula: Modification of Diet in Renal Disease-formula
m24hUNa > 2g/d: Measured 24h-urine-sodium > 2g/day
m24hUNa > 2g/d: Measured 24h-urine-sodium > 2g/day
ND: No diuretic intake
NKF KDOQI: The National Kidney Foundation Kidney Disease Outcomes Quality Initiative
NPV: Negative predictive value
NYHA: New York Heart Association
PM spot urine: Post Meridiem, evening spot urine, before dinner (06:00–08:00 PM)
PPV: Positive predictive value
r: Spearman´s Rho
S: Serum
SD: Standard Deviation
SpotUCrea: Urine-creatinine in spot urine sample
SpotUK: Urine-potassium in spot urine sample
SpotUNa: Urine-sodium in spot urine sample
U: Urine
We24hUNa: Equation for women to predict 24h-urine sodium
We24hUK: Equation for women to predict 24h-urine potassium
WHO: World Health Organization
Z-value: Fisher-Z-value
