# Supplementary figures and images for: Spot urinary sodium in CKD patients: correlation with 24h-excretion and evaluation of commonly used prediction equations
Source: BMC Nephrol. 2024 Jun 27;25:210. doi: 10.1186/s12882-024-03639-2 (PMC11212440; doi:10.1186/s12882-024-03639-2)

Supplement Figure S1

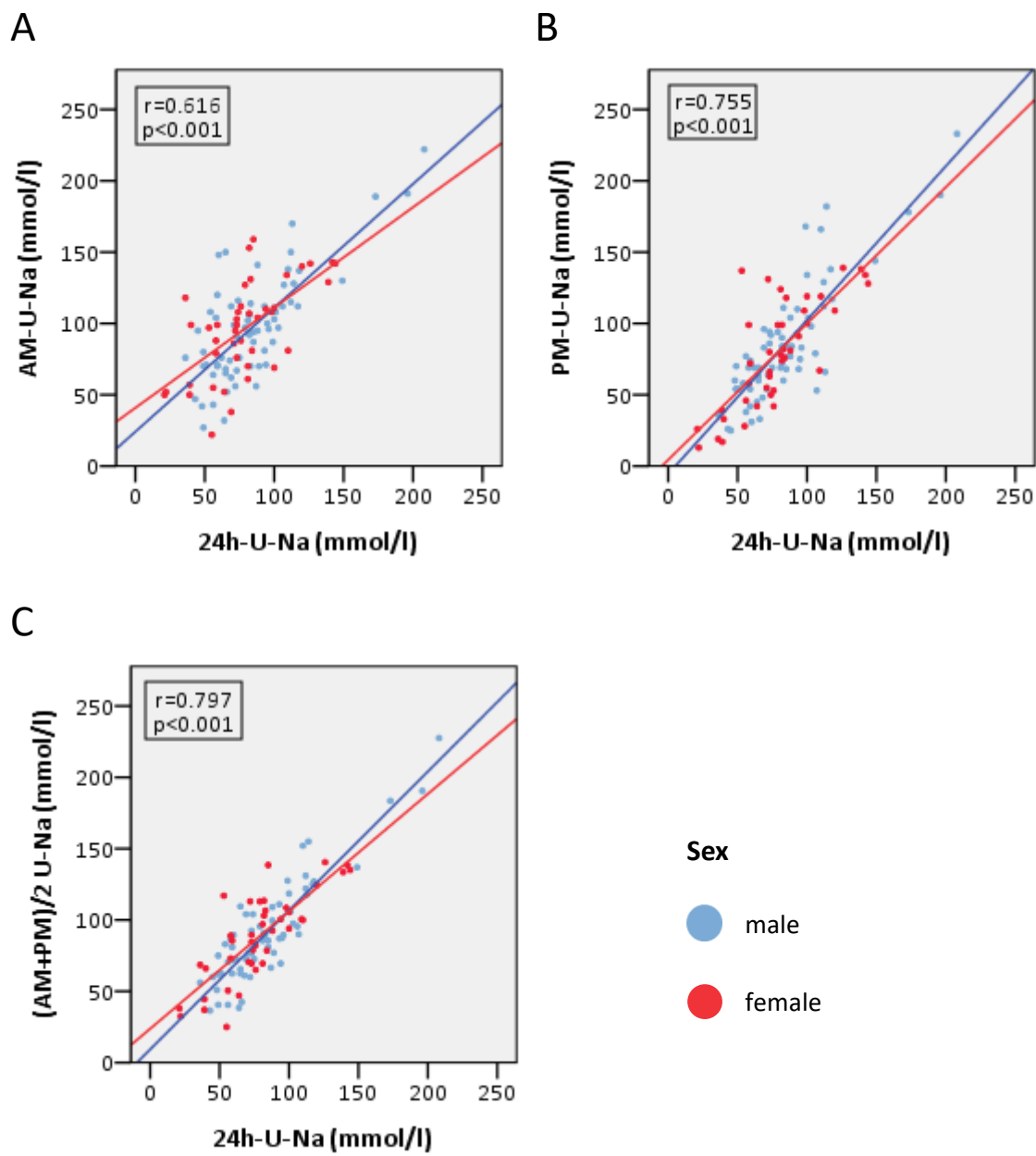

Supplement: Supplementary file 1 — Supplementary Material 1. [file 12882_2024_3639_MOESM1_ESM.pdf]

Supplement Figure S2

A

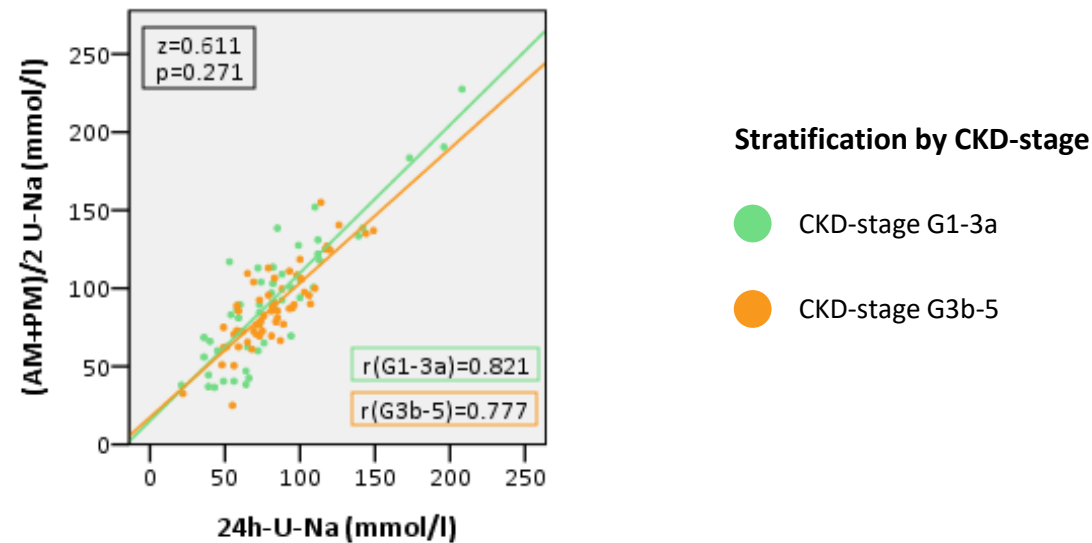

B

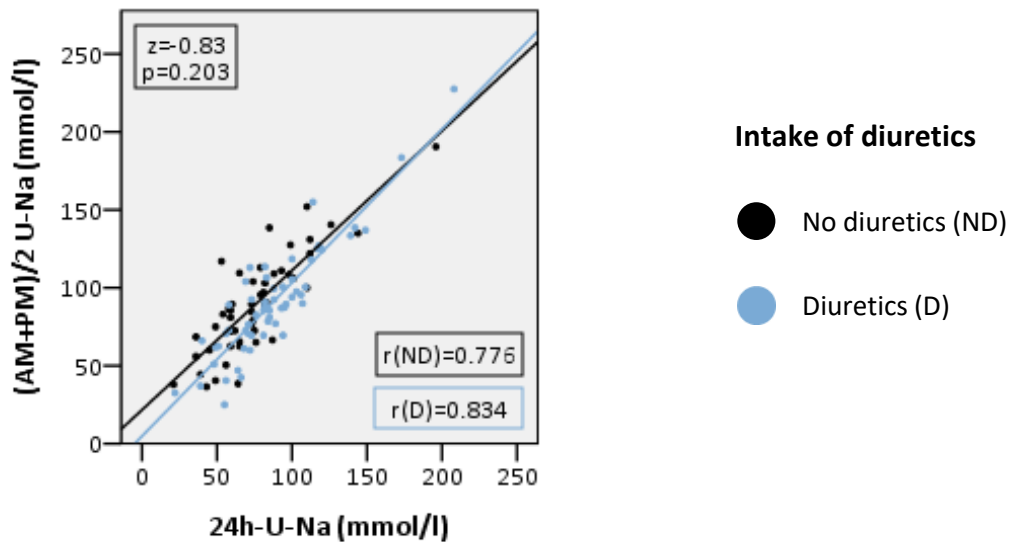

Supplement: Supplementary file 2 — Supplementary Material 2. [file 12882_2024_3639_MOESM2_ESM.pdf]

Supplement Figure S3

A

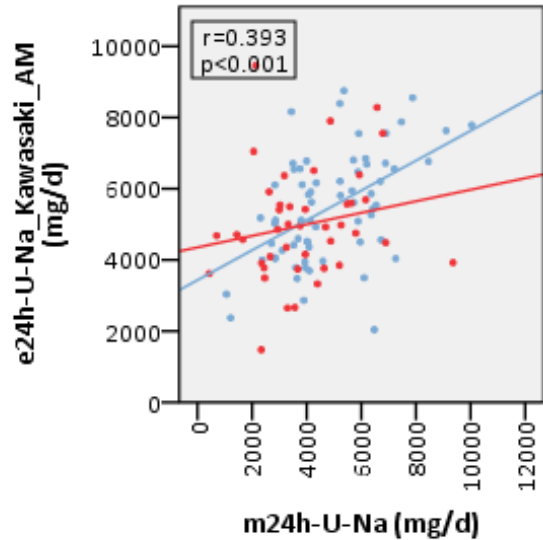

B

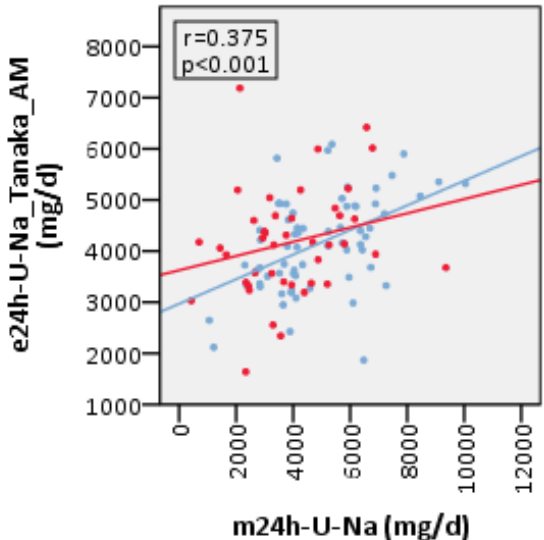

C

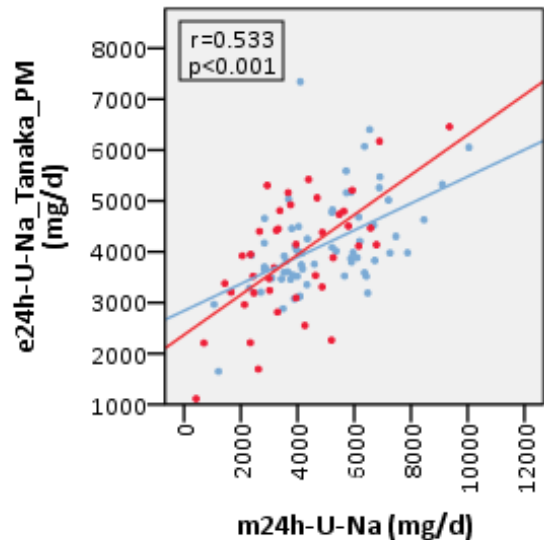

D

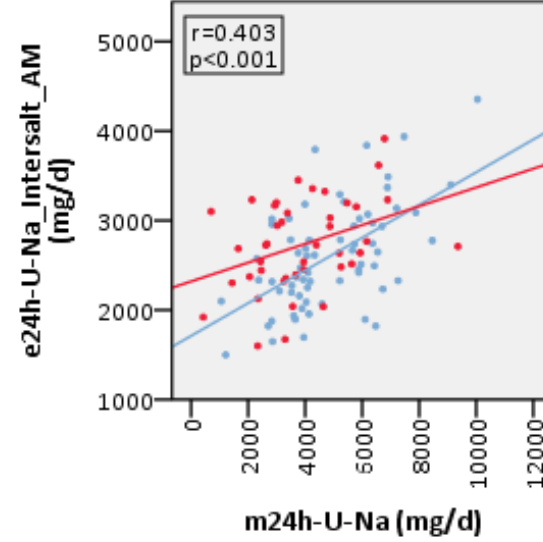

E

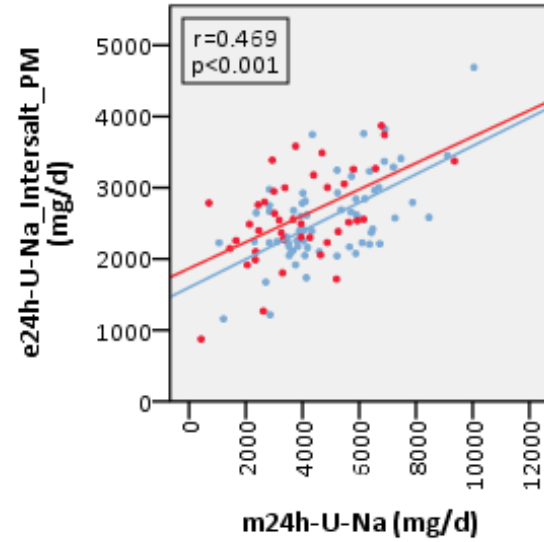

Sex

- male
- female

Supplement: Supplementary file 3 — Supplementary Material 3. [file 12882_2024_3639_MOESM3_ESM.pdf]
