# Supplementary material for: Spot urinary sodium in CKD patients: correlation with 24h-excretion and evaluation of commonly used prediction equations
Source: BMC Nephrol. 2024 Jun 27;25:210. doi: 10.1186/s12882-024-03639-2 (PMC11212440; doi:10.1186/s12882-024-03639-2)
Supplement: Supplementary file 4 — Supplementary Material 4. [file 12882_2024_3639_MOESM4_ESM.docx]

**SUPPLEMENT**

**Supplement figure legends**

**Supplement figure S1: Correlation of U-Na concentration of different spot urine samples with 24h U-Na concentration (r=Spearman´s Rank correlation coefficient)**

**A:** Correlation AM U-Na concentration (mmol/l) with 24h U-Na concentration (mmol/l), AM= morning spot urine sample (second morning urine), n=108, r=0.616, p<0.001

**B:** Correlation PM U-Na concentration (mmol/l) with 24h U-Na concentration (mmol/l), PM=evening spot urine sample, n=107, r=0.755, p<0.001

**C:** Correlation (AM+PM)/2 U-Na concentration (mmol/l) with 24h U-Na concentration (mmol/l), (AM+PM)/2=Average of morning and evening spot urine, n=107, r=0.797, p<0.001; *U-Na=urine sodium, blue: male study participants, red: female study participants*

**Supplement figure S2: Comparison of correlations by CKD stage and intake of diuretics (mmol/l vs. mmol/l)**

**A:** Correlation (AM+PM)/2 U-Na concentration (mmol/l) with 24h U-Na concentration (mmol/l) by CKD stage (G1-3a vs. G3b-5) n(G1-3a)=49, n(G3b-5)=58, r(G1-3a)=0.821, r(G3b-5)=0.777, z=0.611, p=0.271; *p>0.05 and the differences are therefore not significant; green: mild CKD=CKD stage G1-3a, orange: moderate CKD=CKD stage G3b-5*

**B:** Correlation (AM+PM)/2 U-Na concentration (mmol/l) with 24h U-Na concentration (mmol/l) by intake of diuretics

n(ND)=49, n(D)=58, r(ND)=0.776, r(D)=0.834, z=-0.83, p=0.203; *p>0.05 and the differences are therefore not significant; black: no intake of diuretics (ND), blue: intake of diuretics (D)*

*CKD=chronic kidney disease; U-Na=Urine sodium*, r=Spearman´s ranking correlation coefficient), (AM+PM)/2=average morning and evening spot urine; ND=no intake of diuretics; D=intake of diuretics

**Supplement figure S3:** **Comparison of correlations of measured 24h U-Na excretion (m24h-U-Na) with estimated 24h U-Na excretion calculated using established equations**

**A:** Correlation of measured 24h U-Na excretion (m24h-U-Na) with estimated 24h U-Na excretion using the Kawasaki-formula and AM-spot urine (e24h-U-Na_Kawasaki_AM)

n=108, r=0.393, p<0.001

**B:** Correlation of measured 24h U-Na excretion (m24h-U-Na) with estimated 24h U-Na excretion using the Tanaka-formula and AM-spot urine (e24h-U-Na_Tanaka_AM)

n=108, r=0.375, p<0.001

**C:** Correlation of measured 24h U-Na excretion (m24h-U-Na) with estimated 24h U-Na excretion using the Tanaka-formula and PM-spot urine (e24h-U-Na_Tanaka_PM)

n=107, r=0.533, p<0.001

**D:** Correlation of measured 24h U-Na excretion (m24h-U-Na) with estimated 24h U-Na excretion using the Intersalt-formula and AM-spot urine (e24h-U-Na_Intersalt_AM)

n=108, r=0.401, p<0.001

**E:** Correlation of measured 24h U-Na excretion (m24h-U-Na) with estimated 24h U-Na excretion using the Kawasaki-formula and AM-spot urine (e24h-U-Na_Intersalt_PM)

n=107, r=0.469, p<0.001

*U-Na=urine sodium, blue: male study participants, red: female study participants*

**Supplement tables**

| **CGA-classification**  **KDIGO 2012** | | **Albuminuria (mg/g creatinine)** | | |
| --- | --- | --- | --- | --- |
|  |  | **A1 (< 30)**  **number (%)** | **A2 (30-300)**  **number (%)** | **A3 (>300)**  **number (%)** |
| **GFR-categories (ml/min/1.73m²)** | **G1 (≥90)** | 2 (1.9%) | 1 (0.9%) | - |
|  | **G2 (60-89)** | 8 (7.5%) | 4 (3.7%) | 2 (1.9%) |
|  | **G3a (45-59)** | 24 (22.4%) | 4 (3.7%) | 4 (3.7%) |
|  | **G3b (30-44)** | 24 (22.4%) | 11 (10.3%) | 8 (7.5%) |
|  | **G4 (15-29)** | 3 (2.8%) | 5 (4.7%) | 7 (6.5%) |
|  | **G5 (<15)** | - | - | - |

**Supplement table S1: Number of study participants of the 24h urine collection study according to CGA-classification of chronic kidney disease KDIGO-guideline 2012 (G=eGFR according to MDRD-formula, A=albuminuria), n=107**

The colored background shows the risk for mortality and the occurrence of complications according to CKD Prognosis Consortium (KDIGO, 2012): *green=low risk: 10 study participants, yellow=lightly increased risk: 29 study participants, orange=moderately increased risk: 30 study participants, red=high risk: 31 study participants, dark red=very high risk: 7 study participants*

| **Causes of CKD** | **Presumed primary cause** | |
| --- | --- | --- |
|  | **n** | **%** |
| **Diabetic nephropathy** | 17 | 15.7 |
| **Vascular nephropathy** | 10 | 9.3 |
| **Glomerular nephropathy** | 16 | 14.8 |
| **Interstitial nephropathy** | 23 | 21.3 |
| **Other/ primary cause not to be determined/ unknown** | 42 | 39 |
| **Total** | 108 | 100 |

**Supplement table S2: Primary nephrological disease of study participants, n=108**

Primary nephrological disease identified by treating nephrologist. However, the primary disease could not be determined in a considerable proportion of study participants (20.4%)

|  | AM (mmol/l) vs.  24h (mmol/l) | | PM (mmol/l) vs.  24h (mmol/l) | | (AM+PM)/2 (mmol/l) vs. 24h (mmol/l) | |
| --- | --- | --- | --- | --- | --- | --- |
| All | r=0.616  (p<0.001)  n=107 | | r=0.755  (p<0.001)  n=107 | | **r=0.797**  (p<0.001)  n=107 | |
| CKD stage | **G1-3a** | **G3b-5** | **G1-3a** | **G3b-5** | **G1-3a** | **G3b-5** |
|  | r=0.630  (p<0.001)  n=49 | r=0.655  (p<0.001)  n=59 | r=0.793  (p<0.001)  n=49 | r=0.714  (p<0.001)  n=58 | r=0.821  (p<0.001)  n=49 | r=0.777  (p<0.001)  n=58 |
|  | Z=-0.214  p=0.145 | | Z=0.922  p=0.178 | | Z=0.611  p=0.271 | |
| Diuretics | **ND** | **D** | **ND** | **D** | **ND** | **D** |
|  | r=0.505  (p<0.001)  n=49 | r=0.749  (p<0.001)  n=59 | r=0.812  (p<0.001)  n=49 | r=0.718  (p<0.001)  n=58 | r=0.776  (p<0.001)  n=49 | r=0.834  (p<0.001)  n=58 |
|  | Z=-2.084  p=0.019 | | Z=1.148  p=0.125 | | Z=-0.83  p=0.203 | |

**Supplement table S3: Correlation U-Na concentration in different spot urine samples (mmol/l) vs. 24h U-Na concentration (mmol/l) and stratification based on CKD stage and intake of diuretics**

*AM urine = morning spot urine, PM urine = evening spot urine, ((AM+PM)/2)- urine = average morning + evening spot urine, 24h urine = 24h urine collection test, U urine, ND=no intake of diuretics; D=intake of diuretics; bold print is the highest correlation coefficient (Spearman's Rho, r)*

|  | AM (mmol/g Crea) vs.  24h (mmol/d) | | PM (mmol/g Crea) vs.  24h (mmol/d) | | (AM+PM)/2 (mmol/g Crea) vs. 24h (mmol/d) | |
| --- | --- | --- | --- | --- | --- | --- |
| All | r=0.201  (p=0.037)  n=108 | | **r=0.408**  (p<0.001)  n=107 | | r=0.357  (p<0.001)  n=107 | |
| CKD stage | **G1-3a** | **G3b-5** | **G1-3a** | **G3b-5** | **G1-3a** | **G3b-5** |
|  | r=0.115  (p=0.431)  n=49 | r=0.206  (p=0.047)  n=59 | r=0.340  (p=0.017)  n=49 | r=0.429  (p=0.001)  n=58 | r=0.254  (p=0.079)  n=49 | r=0.403  (p=0.002)  n=58 |
|  | Z=-0.47  p=0.319 | | Z=-0.523  p=0.3 | | Z=-0.839  p=0.201 | |
| Diuretics | **ND** | **D** | **ND** | **D** | **ND** | **D** |
|  | r=0.077  (p=0.599)  n=49 | r=0.259  (p=0.047)  n=59 | r=0.394  (p=0.005)  n=49 | r=0.439  (p=0.001)  n=58 | r=0.334  (p=0.019)  n=49 | r=0.351  (p=0.007)  n=58 |
|  | Z=-0.944  p=0.173 | | Z=-0.273  p=0.393 | | Z=-0.096  p=0.462 | |

**Supplement table S4: Correlation U-Na concentration corrected by Creatinine in different spot urine samples (mmol/g Crea) vs. 24h U-Na excretion (mmol/d) and stratification based on CKD stage and intake of diuretics**

*AM urine = morning spot urine, PM urine = evening spot urine, ((AM+PM)/2)- urine = average morning + evening spot urine, 24h urine = 24h urine collection test, U urine, ND=no intake of diuretics; D=intake of diuretics; bold print is the highest correlation coefficient (Spearman's Rho, r)*

| **Comparison of different equations** | | **Kawasaki-equation** | | **Tanaka-equation** | | **Intersalt-equation** | |
| --- | --- | --- | --- | --- | --- | --- | --- |
|  |  | **AM** |  | **AM** | **PM** | **AM** | **PM** |
| **U-Na** | **Spearman‘s Rho** | 0.393 | - | 0.375 | **0.533** | 0.403 | 0.469 |
|  | **p-value** | 0.000 | - | 0.000 | 0.000 | 0.000 | 0.000 |
|  | **mean bias**  **U-Sodium (mg/d)** | -785.6 | - | **+349.0** | +467.0 | +1849.9 | +1934.2 |
|  | **mean bias salt intake (g/d)** | 2.1 | - | **0.9** | 1.2 | 4.8 | 5.0 |
|  | **95% CI (mg/d)**  **(-1.96 SD**  **to**  **+1.96 SD)** | 7455.4  (-4513.3 to +2942.1) | - | 6976.1  (-3139.1 to +3837.0) | **6090.4**  **(-2578.2 to +3512.2)** | 6662.4  (-1481.3 to +5181.1) | 6329.2  (-1204.9 to +5124.3) |

**Supplement table S5: Comparison of different equations in CKD patients for 24h U-Na excretion.**

*AM=morning spot urine=second morning urine (8:00-10:00), PM=evening spot urine, before dinner (18:00-20:00), 95% CI (mg/d) (-1.96 SD to +1.96 SD)=95% confidence interval (mg/d) (-1.96 standard deviation to +1.96 standard deviation), CKD=chronic kidney disease; U=urine*
